# Clinically relevant benzoxaboroles inhibit mRNA processing in *Trypanosoma brucei*

**DOI:** 10.1186/s13104-022-06258-y

**Published:** 2022-12-17

**Authors:** Albina Waithaka, Christine Clayton

**Affiliations:** Heidelberg University Centre for Molecular Biology (ZMBH), Im Neuenheimer Feld 282, D69120 Heidelberg, Germany

**Keywords:** CPSF73, Benzoxaboroles, Tandem affinity purification, mRNA processing

## Abstract

**Objective:**

The cleavage and polyadenylation endonuclease CPSF73 is thought to be the target of the anti-trypanosomal benzoxaboroles AN7973, acoziborole and AN11736. We previously showed that AN7973 inhibits mRNA processing. We here investigated whether the drug candidates acoziborole (for human sleeping sickness) and AN11736 (for nagana in cattle) have the same effect. We also affinity purified tagged CPSF73 from parasites without, or after, AN7973 treatment, and analysed differentially co-purified proteins by mass spectrometry.

**Results:**

AN11736 and acoziborole both inhibited mRNA processing, as demonstrated by decreased levels of spliced mRNAs and accumulation of di- and tri-cistronic mRNAs from the alpha-beta tubulin locus. Treating the cells with AN7973 for 30 min. did not significantly affect the proteins that copurified with CPSF73.

**Supplementary Information:**

The online version contains supplementary material available at 10.1186/s13104-022-06258-y.

## Introduction

**Fig. 1:**
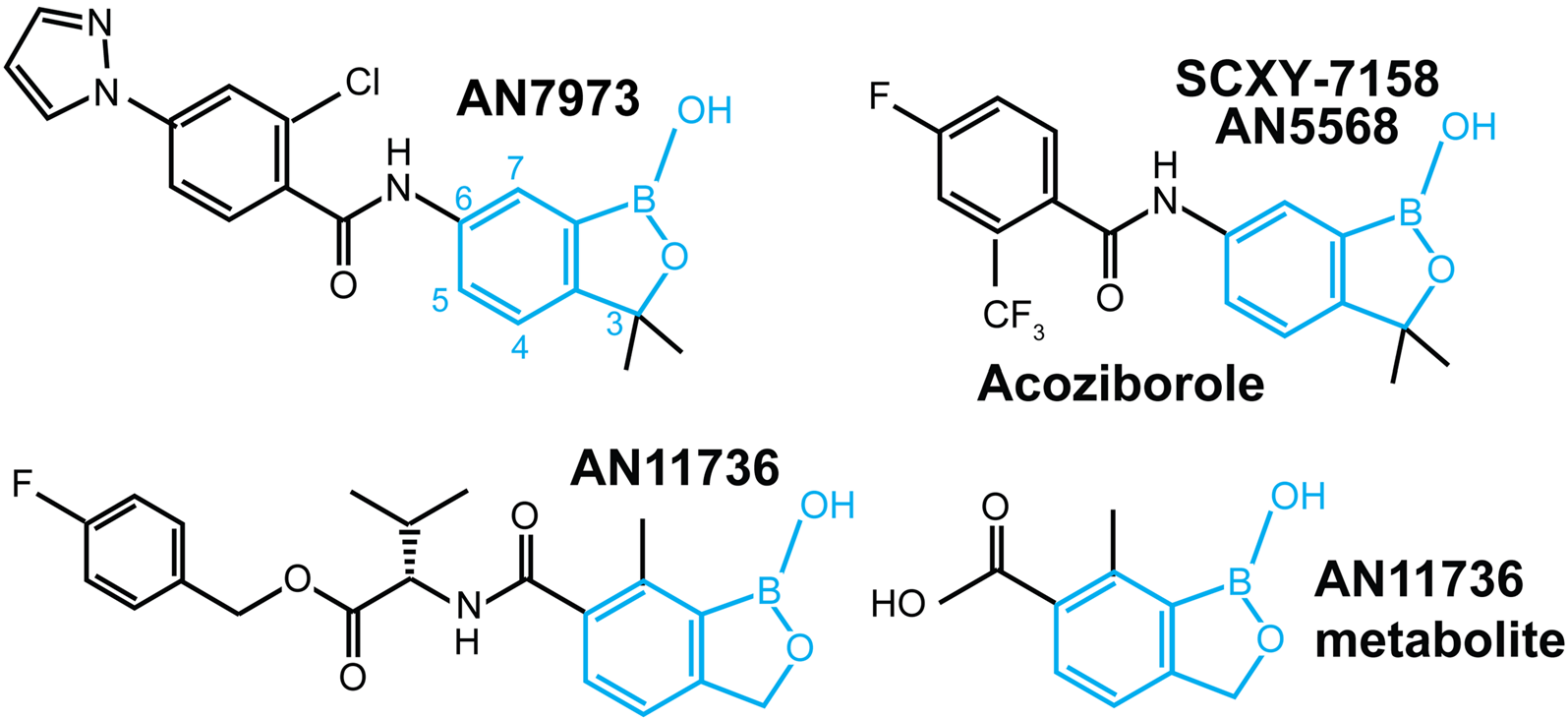
Structures of compounds used. The benzoxaborole core is in blue

The African trypanosomes *Trypanosoma brucei rhodesiense* and *T. brucei gambiense* cause human sleeping sickness, while *T. brucei brucei*, *T. vivax* and *T. congolense* are responsible for African animal trypanosomiasis. Although the human disease is gradually being eliminated as a public health problem [1], infection in animals, particularly cattle, continues to have a serious economic impact [2–5]. Trypanosomiasis treatment relies on chemotherapy [6–8]. Since existing drugs have toxic side-effects and resistance is emerging, new therapies are being sought [7]. The benzoxaboroles acoziborole (SCYX-7158), AN11736 and AN7973 (SCYX-1,608,210) (Fig. 1) are promising anti-trypanosomal compounds. Acoziborole [9] is in phase II/III human clinical trials [10] and AN11736 is a candidate compound for the treatment of animal trypanosomiasis caused by *T. vivax* and *T. congolense* [11]. AN7973 (SCYX-1,608,210) was an early clinical candidate for human trypanosomiasis and might be the basis for a back-up if acoziborole fails [12]. AN11736 is a pro-drug and is active at sub-nanomolar concentrations: it is converted intracellularly into a carboxylate (AN14667), which probably drives drug accumulation [13].

In trypanosomes, mRNAs are transcribed in a polycistronic fashion, and co-transcriptionally processed by *trans* splicing of a 39mer spliced leader (*SL*) to the 5’end [14]. The capped *SL* is derived from the 5′-end of a ~ 140nt precursor, the *SLRNA*. The *trans* splicing reaction is inextricably linked to polyadenylation of the 3′ end of the upstream mRNA [14]. The position of the poly(A) tail is defined by the downstream *trans* splicing reaction [14], and depletion of components of either the *trans* splicing or polyadenylation machineries prevents both splicing and polyadenylation (e.g. [15]). CPSF73 (also called CPSF3) is part of the polyadenylation complex: it catalyses cleavage of the 3′ ends of mRNAs prior to addition of poly(A) tails [16].

We previously showed that AN7973 inhibits trypanosome mRNA *trans* splicing and polyadenylation [17]. Using a primer extension assay for the Y-structure splicing intermediate, we showed that splicing inhibition was detected within an hour. In *T. brucei*, the genes encoding alpha- and beta-tubulin are arranged as an alternating tandem repeat and are co-transcribed from an upstream promoter. By Northern blotting, bi-, tri- and tetra-cistronic tubulin mRNAs are detected within an hour of AN7973 treatment and total mRNA (detected using a spliced leader probe) declined thereafter [17]. Over-expression of CPSF73 increased the IC_50_ of AN7973 [17], and another group obtained similar results for acoziborole and AN11736 [18]. In contrast, for unknown reasons, we found no IC_50_ increase for AN11736 in CPSF73 over-expressing cells. Molecular docking studies suggest that acoziborole and AN7973 bind to the active site of CPSF73 [17, 18].

We here expand these results to fill in two gaps. First, inhibition of mRNA processing by acoziborole has not been demonstrated directly. Secondly, we observed no Y-structure increase after treatment with AN11736, perhaps because our drug sample acted very slowly [17]. We therefore tested both drugs in the Northern blot assay. Secondly, we speculated that binding of AN7973 to CPSF73 might stabilise the polyadenylation and spliceosome complexes, making the mRNA processing machinery unavailable for processing - and potentially also conserving the inter-complex interactions. We therefore tested this by purification and mass spectrometry.

## Materials and methods

Experiments were done using using bloodstream-form Lister 427 strain *T. brucei*. Plasmids and oligonucleotides are listed in Supplementary dataset file 1. EC_50_ determinations and Northern blotting were done exactly as described previously [17]. For the pull-down experiment, the cells were exposed to AN7973 at 10x EC_50_ for 30 min (15 min followed by 13 min centrifugation). The tagged protein was then purified from 1 × 10^9^ cells (at ~ 1 × 10^6^ cells/ml) exactly as described in [19]. Briefly, the protein was allowed to adhere to IgG magnetic beads. After washing, the tagged protein was released using His-tagged tobacco etch virus protease, which was then depleted using nickel-derivatized magnetic beads. We examined four replicates for CPSF73-TAP both with and without AN7973, and for GFP-TAP, one preparation with, and one without, AN7973. The methods for mass spectrometry were as previously described for the RNA-binding protein RBP10 [20]. The samples were run briefly on an SDS polyacrylamide gel and analyzed by mass spectrometry at the ZMBH Core facility. Statistical analysis was performed using Perseus version 1.6.15.0 [21].

### Results and discussion: splicing inhibition

**Fig. 2:**
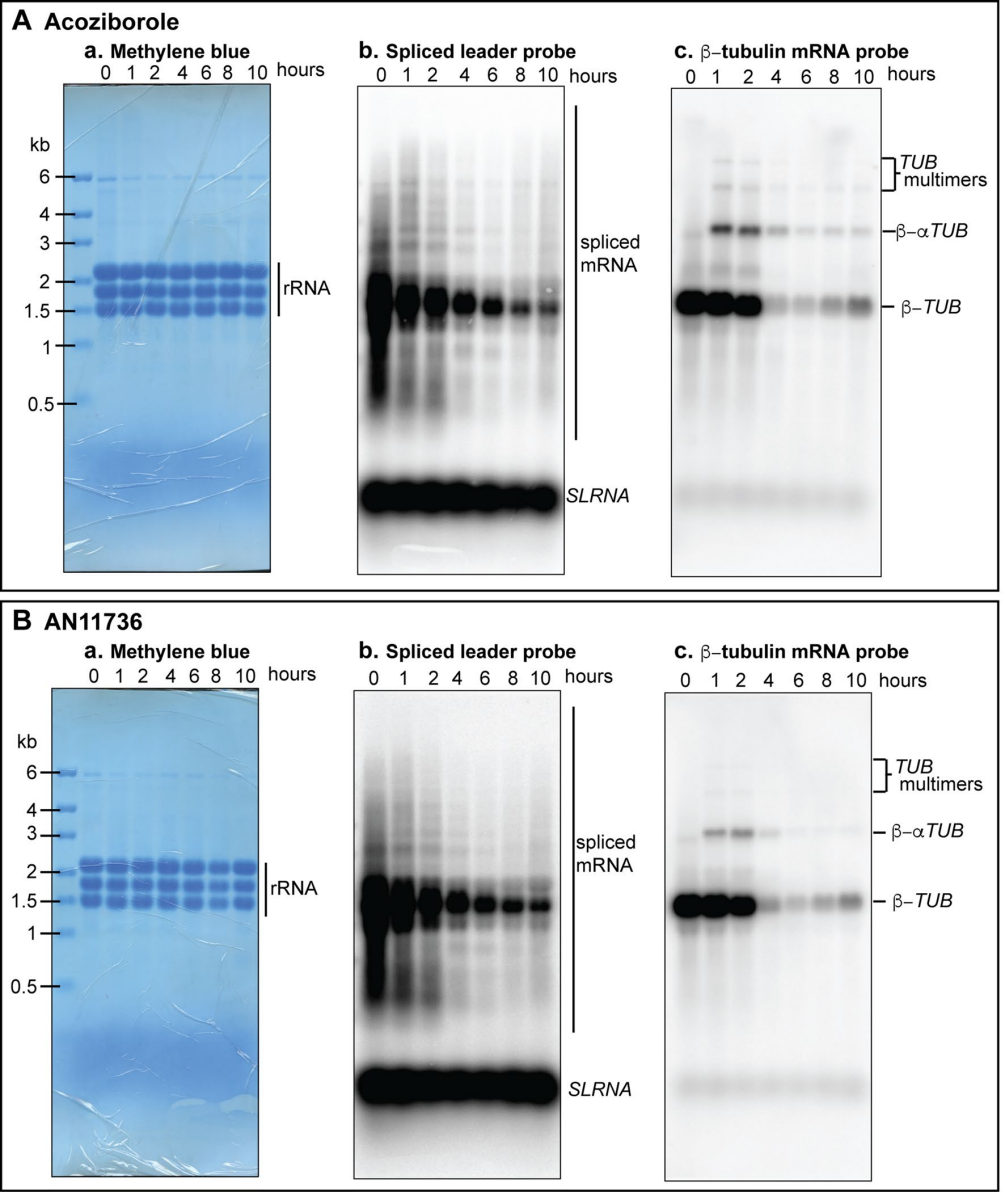
Effect of acoziborole and AN11736 benzoxaboroles on mRNA processing. **A** *T. brucei* bloodstream forms were treated with acoziborole for 10 h. RNA was extracted before adding the compound (0-hour lane) and at the time points indicated on the blots. The RNA was analysed by denaturing gel electrophoresis and Northern blotting. **a** The blots were first stained with methylene blue as loading control. **b** The blot was probed with [^32^P]-labelled oligonucleotide complementary to the spliced leader (*SL*) to detect spliced mRNAs and the 140-nt spliced leader-containing precursor, *SLRNA*. **c** The blot was stripped and hybridised with a ^32^P-labelled probe specific to the mRNA encoding β-tubulin (β-*TUB*). **B** As (**A**) but with AN11763 treatment

We first measured splicing inhibition. Preliminary measurements yielded a sub-nanomolar EC_50_ for AN11736, and an EC_50_ of 512nM for acoziborole. We also confirmed the observation [13] that the carboxylate metabolite of AN11736 (Fig. 1) was much less active than the parent compound: it had no detectable anti-trypanosomal activity at the concentrations tested. To detect splicing inhibition, RNA was collected at different time-points after treatment with 10x EC_50_, which was 6.3 nm for AN11736 (based on published values) and 5.12 μm for acoziborole. Levels of spliced total mRNA and β-tubulin mRNA were then evaluated using Northern blots exactly as previously described [17]. Methylene blue staining, which detects the stable (non-spliced) rRNAs served as the control (Fig. 2A, panel a). Spliced mRNAs were detected by probing the blot with a 39mer oligonucleotide complementary to the spliced leader (*SL*): this detects both processed mRNAs, and the spliced leader RNA (*SLRNA*) substrate for the *trans* splicing reaction. Treatment with acoziborole resulted in gradual reduction in spliced mRNAs (Fig. 2A, panel b). The level of *SLRNA* was probably unaffected (although it is difficult to quantify due to over-exposure), suggesting no substantial inhibition of RNA polymerase II transcription. The blot was then stripped and probed for the β-tubulin (*TUB*) mRNA. As previously observed using AN7973, partially-spliced mRNAs containing two or more tubulin open reading frames accumulated (Fig. 2A, panel c). After 4 h of drug exposure, there was a reduction in total mRNA and *TUB* mRNA. We speculate that at this point, even partial processing is no longer possible and unprocessed mRNA precursors are degraded in the nucleus. Similar results were obtained after treating the cells with AN11736, except that fewer tubulin precursors were detected (Fig. 2B). These findings confirm that AN11736 and acoziborole indeed inhibit mRNA processing.

### Results and discussion: CPSF73-associated proteins

**Fig. 3:**
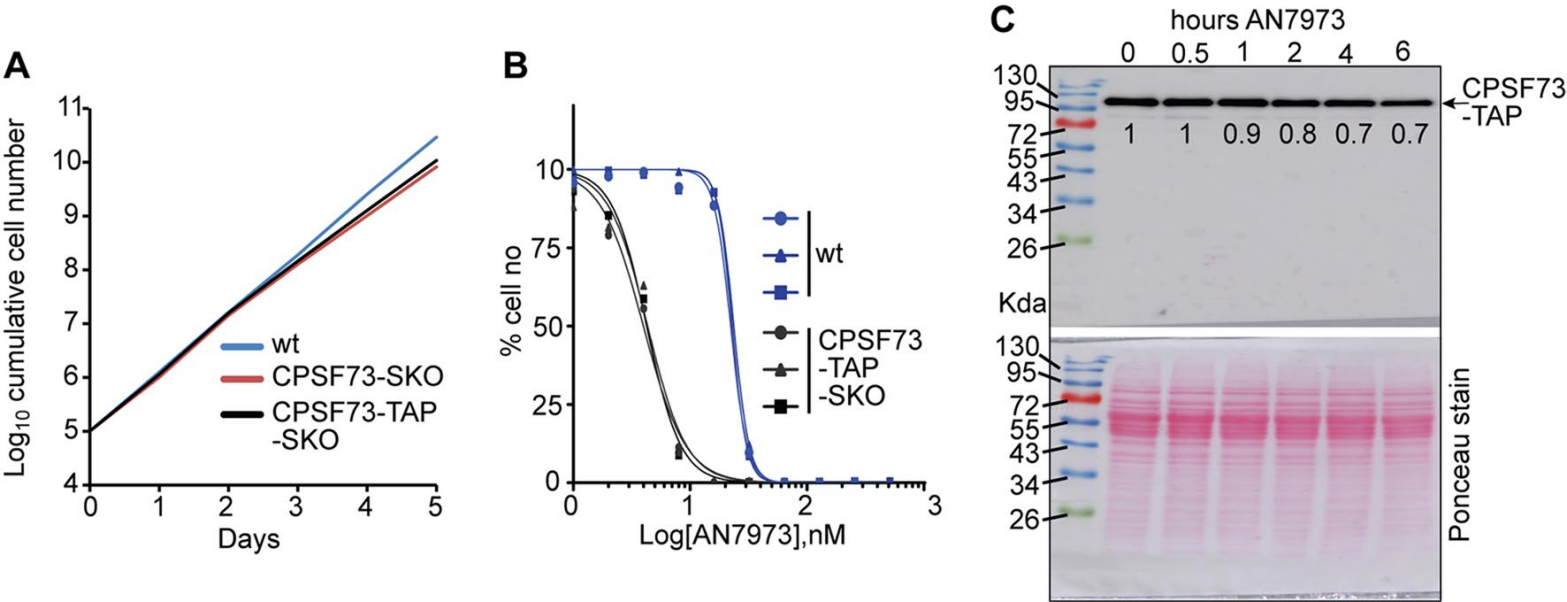
Effect of AN7973 on CPSF73-TAP expression. **A** Growth curve (cumulative cell numbers) showing the viability of cells expressing only TAP-tagged CPSF73 (CPSF73-TAP), or having only a single copy of the *CPSF73* gene (SKO). Cells were counted daily and diluted to maintain densities below 2 × 10^6^/ml. wt = starting cell line. **B** Dose response curves showing the EC_50_ of AN7973 for CPSF73-TAP SKO cells in comparison to wildtype cells. Three technical replicates were analysed. **C** Western blot showing expression of CPSF73-TAP after incubating the cells with AN7973 for 6 h. The values below the bands are the amount of CPSF73-TAP after treatment relative to no treatment. Image below the western blot is ponceau red stain and was used as a loading control

We had speculated that CPSF73 binding might stabilise the polyadenylation complex and its interaction with the splicing machinery. To test this, we compared the proteins that copurified with affinity-tagged CPSF73 with or without prior treatment with AN7973. We first integrated a sequence encoding a tandem affinity purification (TAP) tag downstream of, and in frame with, the *CPSF73* open reading frame (Additional file 1). CPSF73 is an essential gene [15], so to check that the tagged protein was functional, we deleted the wild-type allele and monitored cell growth. In comparison to wildtype cells, the *TAP*-*CPSF73* cells grew slightly slower (Fig. 3A). This might have been a consequence of copy-number reduction since cells with a single wild-type gene also grew at the same slightly slower rate (Fig. 3A). Also, we had replaced the 3’-untranslated region of the tagged allele, which could affect expression. We did not, however, assess CPSF73 protein levels.

Next, we evaluated the cells’ sensitivity to AN7973. For wild-type cells, the average EC_50_ from 3 independent experiments was 22.9 nM (Fig. 3B), agreeing with our previous results [17]. Surprisingly, the CPSF73-TAP cell lines were approximately five times more sensitive to AN7973 than the starting cell line, with an EC_50_ of 4.2nM (Fig. 3B). This might be due to a decreased amount of CPSF73, but this has not been verified. An effect of the tag cannot be ruled out.

Next, we determined the effect of AN7973 on the expression of CPSF73-TAP. Parasites were diluted to 1 × 10^5^ cells/ml and grown for 24 h to final concentration of ~ 1 × 10^6^ cells/ml. The culture was treated with 10x EC_50_ AN7973 for 6 h. Cells were collected for western blot analysis before adding the drug, and at various times thereafter. The amount of CPSF73-TAP was calculated relative to untreated cells (time point 0 h) and the ponceau red stain was used as a loading control. There was a 30% decrease in CPSF73-TAP protein after four hours (Fig. 3C). Previous in vivo [^35^ S]-methionine incorporation assays had showed a dramatic decrease in total protein synthesis only after 4 h of AN7973 exposure [17], but this was almost certainly secondary to loss of mRNA. The half-life of CPSF73 was measured, using pulse-chase and mass spectrometry, to be about 5.5 h [22]. Although the half-life of the *CPSF73-TAP* mRNA is unknown, the decrease in CPSF73-TAP protein after AN7973 treatment could have been due to loss of functional mRNA and therefore, loss of CPSF73 protein synthesis.

To detect effects of AN7973 on protein associations of CPSF73-TAP, cells were either untreated, or exposed to 10x EC_50_ AN7973 for 30 min. Four replicates each were examined. As an additional control, cells expressing GFP-TAP were used, with just two replicates since the composition of the polyadenylation complex is already well known [15]. Tagged proteins were purified and identified by mass spectrometry. Regardless of drug treatment, CPSF73-TAP copurified with members of the polyadenylation complex (Additional file 2): all the CPSF subunits (CPSF7160/100/30/60/73 and Fip1), CstF50, Simplekin and two proteins that co-purify with CPSF160 (Tb927.11.13860 and Tb927.8.4480) [15]. Spliceosome components were not co-purified. The only difference that we noticed after drug treatment was that ubiquitin was detected in three out of four replicates of drug treated samples while it was only detected in one replicate in the untreated cells. However, this difference was not statistically significant, and ubiquitin was also detected in one of the GFP replicates. These results show that AN7973 treatment for 30 min has no significant effect on proteins that co-purify with CPSF73.

## Conclusion

This study confirmed that as expected, acoziborole and AN11736 inhibit mRNA processing. We found no evidence that a 30-min AN7973 treatment affects the composition of the polyadenylation complex or its interaction with the spliceosome.

## Limitations

The Northern blot results were obtained only once, as were the growth curves. The effects of AN7973 on CPSF73-TAP protein levels were measured once and the basis for the possible decrease was not investigated. We did not evaluate the levels of CPSF73-TAP protein in the cell line used for affinity purification relative to native CPSF73 in the starting line, so we do not know why the cells expressing only CPSF73-TAP were more susceptible to AN7973.

## Supplementary Information

**Additional file 1:** Oligonucleotides and plasmids used in the study

**Additional file 2:** Mass spectrometry analysis of proteins that co-purify with CPSF73.

## Data Availability

The mass spectrometry proteomics data have been deposited to the ProteomeXchange Consortium via the PRIDE partner repository with the dataset identifier PXD033513. All other data are in the manuscript.

## Abbreviations

TAP: Tandem affinity purification
*TUB*: Tubulin mRNA
*SLRNA*: Spliced leader RNA precursor
*SL*: Spliced leader
Wt: Wildtype
SKO: Single knockout
